# Multidrug Resistant Enteric Bacterial Pathogens in a Psychiatric Hospital in Ghana: Implications for Control of Nosocomial Infections

**DOI:** 10.1155/2017/9509087

**Published:** 2017-09-06

**Authors:** Kwabena O. Duedu, George Offei, Francis S. Codjoe, Eric S. Donkor

**Affiliations:** ^1^Department of Biomedical Sciences, School of Basic and Biomedical Sciences, University of Health and Allied Sciences, Ho, Ghana; ^2^Department of Medical Laboratory Science, School of Biomedical and Allied Health Sciences, University of Ghana, Accra, Ghana; ^3^Department of Medical Microbiology, School of Biomedical and Allied Health Sciences, University of Ghana, Accra, Ghana

## Abstract

Enteric bacteria are commonly implicated in hospital-acquired or nosocomial infections. In Ghana, these infections constitute an important public health problem but little is known about their contribution to antibiotic resistance. The aim of the study was to determine the extent and pattern of antibiotic resistance of enteric bacteria isolated from patients and environmental sources at the Accra Psychiatric Hospital. A total of 265 samples were collected from the study site including 142 stool and 82 urine samples from patients, 7 swab samples of door handle, and 3 samples of drinking water. Enteric bacteria were isolated using standard microbiological methods. Antibiograms of the isolates were determined using the disc diffusion method. Overall, 232 enteric bacteria were isolated.* Escherichia coli* was the most common (38.3%), followed by* Proteus* (19.8%),* Klebsiella* (17.7%),* Citrobacter* (14.7%),* Morganella* (8.2%), and* Pseudomonas* (1.3%). All isolates were resistant to ampicillin but sensitive to cefotaxime. The resistance ranged from 15.5% to 84.5%. Multidrug resistance was most prevalent (100%) among isolates of* Proteus* and* Morganella* and least prevalent among isolates of* Pseudomonas* (33.3%). Multidrug resistance among enteric bacteria at the study hospital is high and hence there is a need for screening before therapy to ensure prudent use of antibiotics.

## 1. Introduction

Psychiatric hospitals are specialized settings for the treatment of mental disorders. However, patients admitted in these hospitals are at a higher risk of acquiring nosocomial infections, which are the leading causes of death and increased morbidity for hospitalized patients [1–4]. In general, the sources of nosocomial infections can be categorized into environmental factors (such as the quality of water used at the hospital, inanimate objects, and architectural design), patient-related factors, iatrogenic factors (surgery and invasive procedures, devices and equipment, and antibiotic use), crowdedness at the hospital, and the hands of healthcare workers. Patients are at minimal risk if they have no significant underlying disease, have an intact immune system, and have not undergone any invasive procedure. High risk is assigned to patients who are susceptible to disease for a variety of reasons, including decreased immune function, low nutritional status, and mental disorders [5, 6].

Enteric bacterial pathogens are commonly implicated in nosocomial infections, particularly in environments where poor hygiene prevails [2, 4]. These organisms can live on inanimate surfaces for as long as seven days to several months in the hospital environment [7, 8]. They can also contaminate the drinking water and food of patients in the hospital [9, 10]. There is evidence that some insects, particularly cockroaches, could serve as vectors for dissemination of enteric pathogens in the hospital environment [11–13]. The common enteric bacterial pathogens implicated in nosocomial infections include* Escherichia coli*,* Pseudomonas*,* Klebsiella*, and* Salmonella* [2, 4].

Though antibiotic drugs have reduced the burden of common bacterial diseases and become essential for many medical interventions [14]; in recent times pathogens have become resistant to such drugs at an alarming rate. Drug resistant bacteria have been reported to cause about 20–70% of nosocomial infections [15]. The inappropriate use of antibiotics is a major contributor to the increase in drug resistant strains of bacteria, and, coupled with the natural selection and exchange of genetic resistance elements among microorganisms, drug resistance has emerged as a worldwide problem [16, 17]. Multidrug resistant strains of enteric bacteria such as* Salmonella*,* Acinetobacter*, and* Pseudomonas* have been implicated in outbreaks in both developed and developing countries [18, 19]. Though a global problem, drug resistance in bacteria poses a greater threat in the developing world where morbidity of bacterial diseases is much higher and treatment options are limited [20–22].

In Ghana, nosocomial infections constitute an important public health problem but little is known about these infections in relation to antibiotic resistance. The few studies on antibiotic resistance of nosocomial pathogens in Ghana seemed to have focused on a few causative organisms particularly* Staphylococcus aureus* [23–26], and there is hardly any data on enteric organisms. Additionally, previous studies on nosocomial infections in Ghana and many countries have focused on general hospital wards, and there is no data on special wards such as psychiatric hospitals. In Ghana, psychiatric wards are generally characterized by poor sanitary conditions; coupled with overcrowding and other factors, enteric organisms could be readily disseminated in the environment of these wards. The risk of enteric organisms in psychiatric wards in Ghana highlights the need for the current study, which was aimed at determining the extent and pattern of antibiotic resistance of enteric bacteria isolated from patients and environmental sources in a Ghanaian psychiatric hospital.

## 2. Materials and Methods

### 2.1. Study Area and Sampling

The study involved convenience sampling of patients and the environment at the Accra Psychiatric Hospital located in Accra (capital city of Ghana) from January to June, 2012. The hospital is a leading mental health facility serving the city of Accra, its environs, and the entire southern section of Ghana. Two hundred (200) inmates were randomly selected at the Accra Psychiatric Hospital wards, namely, the Special Ward, Wards C1 and C2, the Male Admission Ward, the Bank for Housing and Construction (BHC) Ward, the Male Infirmary Ward, the female Geriatric Ward, and the Female Infirmary Ward. Apart from hospitalisation for psychiatric reasons, the study participants were apparently healthy. Stool and/or urine samples were collected aseptically from the study participants who consented to provide the samples. Their drinking water sources were also sampled, while swabs samples were collected from door handles in the hospital wards. Overall, 265 samples were collected including 142 stool samples, 82 urine samples, 7 swab samples of door handle, and 3 samples of drinking water. The samples collected were transported immediately to the Bacteriology Laboratory of the School of Biomedical and Allied Health Sciences of University of Ghana for analysis.

### 2.2. Laboratory Analysis

#### 2.2.1. Isolation and Identification of Bacteria

Stool samples were inoculated in Selenite F broth and subcultured onto deoxycholate agar. Urine samples were inoculated on Cysteine Lactose Electrolyte Deficient agar. Swab and water samples were inoculated on blood agar and MacConkey agar. The inoculated bacteriological media were incubated between 18 and 24 hours at 37°C. Bacterial isolates were identified based on colonial morphology, Gram stain, and a battery of biochemical tests.

#### 2.2.2. Antimicrobial Susceptibility Test

Antimicrobial susceptibility tests of bacterial isolates were done by the disc diffusion method according to the European Committee on Antimicrobial Susceptibility Testing (EUCAST) standards [27]. A minimum of four to five pure colonies of the test organism from an overnight growth was transferred into peptone water and the suspension was adjusted to a density of 0.5 McFarland turbidity standard. Optimally, within 15 minutes after adjusting the turbidity of the inoculum suspension, a sterile cotton swab was dipped into the adjusted suspension. The swab was rotated several times and pressed firmly on the inside wall of the tube above the fluid level to remove excess inoculum from the swab. The dried surface of a Mueller-Hinton agar plate was inoculated by streaking the swab over the entire sterile agar surface. This procedure was repeated by streaking two more times, rotating the plate approximately 60° each time to ensure an even distribution of inoculum. The lid of the agar plate was left ajar for 3 to 5 minutes, but no more than 15 minutes, to allow for any excess surface moisture to be absorbed before applying the drug impregnated disks.

Antimicrobial discs of 10 *μ*g ampicillin, 30 *μ*g tetracycline, 10 *μ*g gentamicin, 30 *μ*g cefuroxime, 30 *μ*g chloramphenicol, 30 *μ*g cefotaxime, 30 *μ*g tetracycline, 30 *μ*g amikacin, and 25 *μ*g of cotrimoxazole were applied firmly to the surface of the inoculated agar plate. Urine antimicrobial discs were also used for the bacterial isolates from the urine samples. Within 15 minutes after the discs were applied, the plates were inverted and incubated aerobically at a temperature of 35°C for 18–24 hrs. Quality control testing was done by using* Escherichia coli* ATCC25922 strain for validation of the susceptibility testing process. Zone diameters around the antibiotic discs were measured and classified as sensitive or resistant based on the EUCAST break point system [27].

### 2.3. Data Analysis

Data were analyzed using the IBM SPSS Statistics version 23 (IBM Corporation, Armonk, NY). Descriptive analysis including frequencies and percentages (prevalence) was computed for the various enteric bacteria isolated from the specimens. Prevalence of an enteric bacterial organism was computed as a proportion of the total number of enteric isolates. Analyses of frequencies and percentages were done for resistant and multidrug resistance isolates. Multidrug resistance was defined as resistance to three or more classes of antimicrobial agents.

## 3. Results

Overall, 237 (88.5%) of the 265 samples collected were culture positive and the enteric bacteria isolated are shown in Table 1.* Escherichia coli* was isolated from all the specimen types and was most prevalent in urine (58.6%).* Proteus* and* Klebsiella* were isolated from only urine and stool samples;* Proteus* had similar prevalence in stool (20.4%) and urine (20.7%), while* Klebsiella* was more prevalent in urine (20.7%).* Citrobacter* and* Morganella* were isolated from only stool specimens with prevalence of 23.9% and 13.4%, respectively.* Pseudomonas* was isolated from only door handles at a prevalence of 60%. Overall,* E. coli* was the most common organism isolated (38.3%), followed by* Proteus* (19.8%),* Klebsiella* (17.7%),* Citrobacter* (14.7%),* Morganella* (8.2%), and* Pseudomonas* (1.3%).

**Figure 1:**
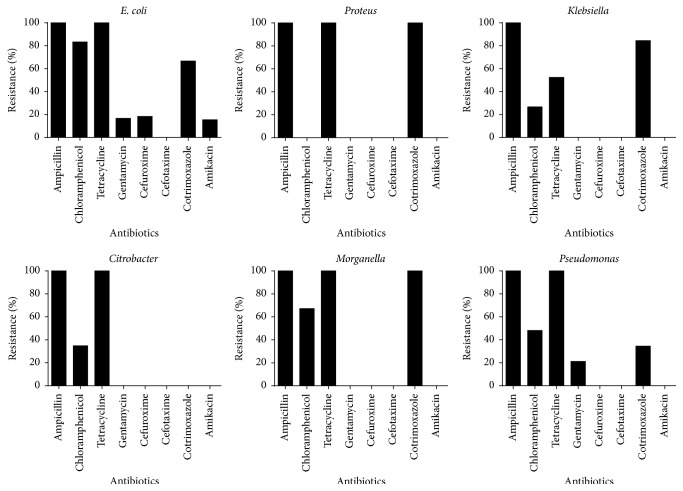
Antibiogram of six enteric bacterial organisms isolated at the Accra Psychiatric Hospital.

Antimicrobial susceptibility testing results of the various enteric bacteria isolated are shown in Figure 1. All isolates were resistant to ampicillin and tetracycline except for* Klebsiella *where only 52% were resistant to tetracycline. Various degrees of resistance were observed for chloramphenicol for all the isolates with the exception of* Proteus.* The* E. coli* isolates were resistant to ampicillin and tetracycline but sensitive to cefotaxime. High* E. coli* resistance was also observed for chloramphenicol (83.3%) and cotrimoxazole (66.7%). All the* Proteus* isolates were resistant to ampicillin, tetracycline, and cotrimoxazole but sensitive to chloramphenicol, gentamycin, cefuroxime, and cotrimoxazole. All the* Klebsiella* isolates were resistant to ampicillin but sensitive to gentamycin, cefuroxime, cefotaxime, and amikacin; high percentage resistance of* Klebsiella* was also observed for tetracycline (52.38%) and cotrimoxazole (84.45%). All the* Citrobacter* isolates were sensitive to the antibiotics tested except chloramphenicol, ampicillin, and tetracycline, which had percentage resistance of 34.8%, 100%, and 100%, respectively. Overall, all the isolates tested were resistant to ampicillin but sensitive to cefotaxime. As shown in Table 2, multidrug resistance was most prevalent among isolates of* Proteus* (100%) and* Morganella* (100%) and least prevalent among isolates of* Pseudomonas* (33.3%).

## 4. Discussion

In this study, antibiotic resistance among enteric bacteria at a psychiatric hospital in Ghana was investigated. The patterns of antibiotic resistance of the various enteric bacteria were similar for six of the eight drugs tested including ampicillin, tetracycline, gentamicin, cefuroxime, cefotaxime, and amikacin. This may be due to the similarity of structural and genetic resistance properties that are shared by this family of bacteria. The presence of an outer membrane in enteric bacteria (Gram negative bacteria) generally excludes antibiotics from penetrating the cell [28]. Additionally, these organisms have a great facility for exchanging genetic material (DNA) among strains of the same species and even among different species [29].

Extremely high percentage resistance to ampicillin and tetracycline was observed, which concurs with previous studies on enteric organisms in Ghana [30, 31]. In enteric organisms, tetracycline resistance is mediated by* tet *(A) and* tet* (B) genes that encode efflux proteins. Resistance to ampicillin and other beta-lactam drugs in these organisms is mediated by *β*-lactamases. The introduction of new *β*-lactams with different activity spectra has led to a selection of different genes and mutations that confer resistance to these drugs in enteric bacteria [23, 32]. Epidemiological evidence suggests that the increasing resistance prevalence of tetracycline, ampicillin, and many other antibiotics is directly linked to their usage. In Ghana, tetracycline and ampicillin have been on the market for a very long time; this, coupled with the high rates of self-medication in the country [30, 31], contributes significantly to the high bacterial resistance observed in this study. The lower prevalence of resistance observed for cefuroxime, cefotaxime, and amikacin in this study is probably because these antibiotics have been on the market for relatively shorter period and may not have been subjected to high usage like ampicillin and tetracycline. In addition, some of these drugs such as cefotaxime are expensive and this tends to limit their usage in Ghana where the incomes of many people are still low.

The data of this study suggests that multidrug resistance may be widespread among enteric bacterial organisms in Ghana. This is not surprising, as enteric bacteria tend to be associated with mobile genetic elements such as plasmids and transposons, which usually carry multiple resistance genes [33]. The enteric organisms investigated in this study have been implicated in various nosocomial infections, and their high levels of resistance have important implications for treatment of such infections in the study setting. For example,* E. coli*, which showed multidrug resistance prevalence of 83.3%, is a leading cause of nosocomial urinary tract infection [34–36]. Similarly,* Klebsiella*, which is a common cause of blood stream infections in the hospital setting [37–39], showed high multidrug resistance prevalence of 51.2%. In Ghana, antibiotic treatment options are relatively limited and, therefore, the high prevalence of multidrug resistance observed could have a significant impact on public health. The gut is the main source of enteric bacteria and therefore the risk of dissemination of multidrug resistance enteric bacteria from this source is probably high. The key risk factors for dissemination include dependency for toileting, diarrhea, and living in countries where hygiene is poor [40–42]. Consequently, the very low compliance with hand hygiene rules in most hospitals in the developing world is a major obstacle to preventing the cross-transmission of multiresistant enteric bacteria.

## 5. Conclusion

This study concludes that multidrug resistance is highly prevalent among enteric bacterial organisms in the study hospital. This probably reflects the trend in other hospitals in Ghana. The study has exposed an important public health problem regarding antibiotic resistance of enteric bacteria related to the hospital environment. Two recommendations to begin to address this problem are (a) creation of more awareness among clinicians and other healthcare professionals of the potential interventions, (b) surveillance of antibiotic resistance among enteric pathogens, and (c) further research to more clearly define the problem and how it can be addressed. In particular, there is the need to use molecular methods to further investigate antibiotic resistance of enteric pathogens.

## Figures and Tables

**Table 1:** Enteric bacterial organisms isolated from various specimens at the Accra Psychiatric Hospital.

Enteric organism	Type of specimen				Total isolates *n* (%)
	Stool *n* (%)	Urine *n* (%)	Door handle *n* (%)	Water *n* (%)	
*E. coli*	36 (25.4)	48 (58.6)	2 (40.0)	3 (100.0)	89 (38.3)
*Proteus*	29 (20.4)	17 (20.7)	0 (0.0)	0 (0.0)	46 (19.8)
*Klebsiella*	24 (16.9)	17 (20.7)	0 (0.0)	0 (0.0)	41 (17.7)
*Citrobacter*	34 (23.9)	0 (0.0)	0 (0.0)	0 (0.0)	34 (14.7)
*Morganella*	19 (13.4)	0 (0.0)	0 (0.0)	0 (0.0)	19 (8.2)
*Pseudomonas*	0 (0.0)	0 (0.0)	3 (60.0)	0 (0.0)	3 (1.3)
*Total isolates*	142 (100.0)	82 (100.0)	5 (100.0)	3 (100.0)	232 (100.0)

Number outside parentheses indicates number of individual isolates (*n*); number in parentheses indicates the corresponding proportion of isolates in percent (%).

**Table 2:** Prevalence of multidrug resistant isolates among enteric bacterial organisms at the Accra Psychiatric Hospital.

Enteric bacterial organism	*n*	%
*Proteus*	46	100
*Morganella*	19	100
*Escherichia coli*	74	83.3
*Klebsiella*	21	51.2
*Citrobacter*	11	33.4
*Pseudomonas*	1	33.3
